# Oral cholera vaccine coverage in Goma, Democratic Republic of the Congo, 2022, following 2019–2020 targeted preventative mass campaigns

**DOI:** 10.1016/j.jvacx.2024.100555

**Published:** 2024-09-12

**Authors:** Emily Briskin, Stéphane Hans Bateyi Mustafa, Rachel Mahamba, Deka Kabunga, Janvier Kubuya, Klaudia Porten, Epicentre-MSF DRC cholera working group, Laurent Akilimali, Placide Okitayemba Welo, Anaïs Broban

**Affiliations:** a Epicentre, Paris, France; b Programme Elargi de Vaccination, North Kivu, Democratic Republic of the Congo; c Provincial Health Division, North Kivu, Democratic Republic of the Congo; d Epicentre, Goma, Democratic Republic of the Congo; e National Program for the Elimination of Cholera and the Control of Other Diarrheal Diseases (PNECHOL-MD), Democratic Republic of the Congo

**Keywords:** Cholera, Targeted vaccination, Oral cholera vaccine, Vaccine coverage survey, Democratic Republic of the Congo

## Abstract

**Background:**

In 2019–2020, preventative Oral Cholera Vaccine campaigns were conducted in 24/32 non-contiguous health areas of Goma, DR Congo. In August 2022, we measured coverage and factors potentially influencing success of the delivery strategy.

**Methods:**

We used random geo-sampled stratified cluster survey to estimate OCV coverage and assess population movement, diarrhea history, and reasons for non-vaccination.

**Results:**

603 households were visited. Coverage with at least one dose was 46.4 % (95 %CI: 41.8–51.0), and 50.1 % (95 %CI: 45.4–54.8) in areas targeted by vaccination compared to 26.3 % (95 %CI: 19.2–34.9) in non-targeted areas. Additionally, 7.0 % of participants reported moving from outside Goma since 2019, and 5.4 % reported history of severe diarrhea. Absence and unawareness were the main reasons for non-vaccination.

**Conclusion:**

Results suggest that targeting non-contiguous urban areas had a coverage-diluting effect. Targeting entire geographically contiguous areas, adapted distribution, and regular catch-up campaigns are operational recommendations to reach higher coverages arising from the study.

## Highlights

• Oral cholera vaccine (OCV) coverage in Goma urban area was 46.3 % two years post-campaign.
• Micro-targeting of non-contiguous urban zones led to dilution of coverage.
• Population movements and displacement created challenges for maintaining coverage.
• Door to door strategies should account for people being away during the day.

## Background

Cholera remains an important public health issue worldwide and is recently resurging, including in countries that previously had gone many years without outbreaks [1], [2]. The Oral Cholera Vaccine (OCV) is one of the cholera control tools, individuals aged 1 and older being eligible, with two doses administered 14 days apart. Two doses have been shown to provide about 58 % protective efficacy and 76 % effectiveness over 2 years, starting to decline after about 2 years, with few reliable estimates of long-term protective effect available. Efficacy appears to be lower among children under five [3], [4]. Previous mass campaigns have shown good OCV safety and acceptability [5], [6], [7].

The World Health Organization (WHO), following Ending Cholera: A Global Roadmap to 2030, developed by the Global Task Force on Cholera Control [3], [8] recommends reactive and preventative cholera control integrated strategies, including water and sanitation and OCV mass campaigns. However, limited availability has historically constrained OCV use for large-scale cholera control, and information on the optimal strategies for such vaccination campaigns remains limited [9].

Goma, capital of the North-Kivu Province, is the fourth-largest city in Democratic Republic of the Congo and is composed of health areas (HA) across three health zones (Karisimbi, Goma, and Nyiragongo). The city is affected by recurrent natural disasters and population displacement related to armed conflict, and is considered a cholera hotspot with recurrent outbreaks [10], [11]. As part of the national Multi-Sectoral Plan for Cholera Elimination [12], a number of cholera-endemic zones of the country, including Goma city, which is likely seeding cases across the region, were selected for targeted preventative OCV mass campaigns. Based on routine surveillance data during pre-vaccination time, several geographically non-contiguous HA were selected for two-dose campaigns in May/October 2019, then in January/June 2020, and one additional HA was targeted for a single-dose campaign in June 2020. In this challenging context (population uncertainties, frequent movements, limited resources, dense neighborhoods), the campaigns used a door-to-door strategy, targeting all people aged one year and above. Post-campaigns coverage rapid random assessments in targeted areas resulted in levels ranging from 73 % to 98 % (Supplementary Data) [13], though post-campaign overall city coverage, as well as population-level protection achieved by targeted OCV campaigns, remained unclear. In August 2022, as part of a larger project evaluating the impact of OCV campaigns in endemic zones (Goma and Bukama, Haut-Lomami), and before large displacements of North-Kivu population to Goma occurred (late 2022), an urban survey was conducted to measure current overall OCV coverage levels and factors potentially affecting coverage.

## Methods

**Fig. 1:**
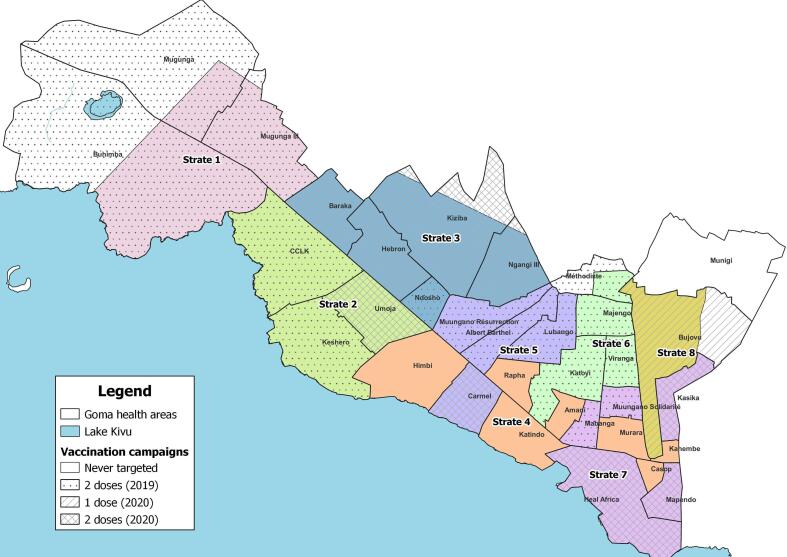
History of Oral Cholera Vaccination Campaigns, 2019–2020 by number of does (hashing), and strata (colors) used for vaccination coverage survey, 2022, Goma, Democratic Republic of the Congo.

We conducted a stratified cluster survey based on standard MSF protocol (Supplementary Data) [14], [15]. Goma city’s HA and surrounding *peri*-urban area were divided into eight strata, according to number of OCV doses received, population density and geographical continuity (Supplementary Table 1). Assuming a coverage of 50 %, precision of 8 %, α = 5 %, and a design effect of 2, we surveyed 20 clusters of 4 households by strata. For security reasons, the survey was restricted to urban areas, cutting out some unstable sub-urban zones (Fig. 1). Random geo-spatial points were identified using GIS software within each stratum. Only geo-points with a roof within a 50 m radius were kept (using satellite images checks), and other points selected if necessary. Clusters were interviewed around each geo-point, systematically selecting the fifth house on the left of the first included house, until cluster was complete. When several households lived under the same roof, one was randomly selected among eligible ones. All individuals aged one and above at survey time and living in the survey area were eligible for inclusion. Heads of households or their representative were interviewed about the vaccination status of all household members (people who slept under the same roof the previous night). Unavailable households were replaced and not revisited.

We used KoboCollect software to collect data on demographic characteristics, history of vaccination, migration and severe watery diarrhea (3 watery stools in 24 h) since January 2019 (Supplementary Table 2). Using R software (version 4.1.3, R4EPI/epikit package) we calculated population-weighted proportion of the participants’ according to demographic profiles, and calculated vaccination coverage levels and confidence intervals. Participants were considered moving from outside Goma city when they moved from other HA than included in the survey strata (Fig. 1). After excluding participants with unknown vaccination status, we assessed vaccination status’ association with age group and sex using univariate logistic regression. Association with other variables was assessed using multivariate logistic regression, adjusting on age group and sex.

This survey was conducted in accordance with the ethical guidelines of the Council for International Organizations of Medical Sciences [16]. The protocol was reviewed and approved by the Ethical Committee of the University of Kinshasa School of Public Health (ESP/CE/106/2022, 11/08/2022), and by the MSF Ethics Review Board (ID 2070, 20/07/2022).

## Results

The survey was carried out between 22 and 26 August 2022. Surveyors visited 603 households (3,352 individuals, of which 94 had unknown vaccination status). Among respondents, 52.0 % were female, and the median age was 20 years (IQR 10–31). Seven households refused participation.

### Vaccine coverage

**Fig. 2:**
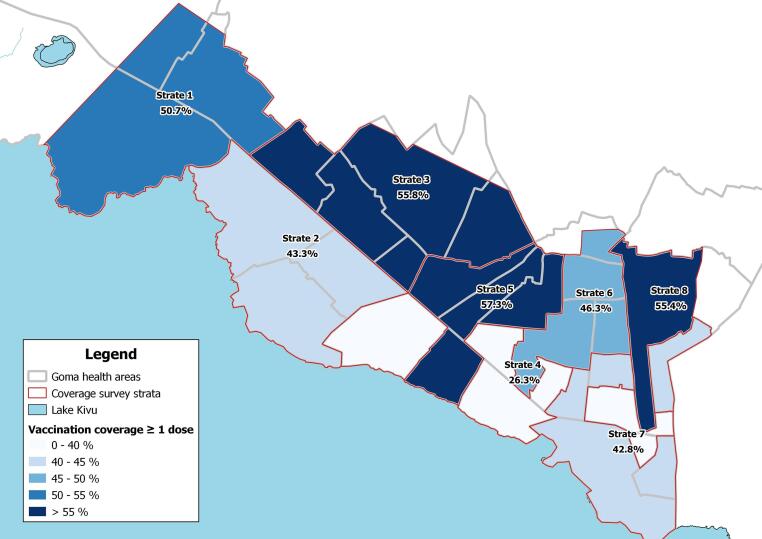
Coverage with at least one dose of oral cholera vaccine (colored), by survey strata, Goma, Democratic Republic of the Congo, August 2022.

Overall coverage with at least one dose was 50.1 % (95 %CI: 45.1–55.0)) in vaccinated areas, compared to 26.3 % (95 %CI: 19.2–34.9) in non-vaccinated areas (Table 1, Fig. 2). Two-dose coverage in vaccinated areas was 13.5 % (95 %CI: 10.2–17.5). Among participants reporting being vaccinated, only 1.2 % presented the physical vaccination card. The main reported reasons for non-vaccination among unvaccinated participants were unawareness (36 %) and absence (24 %) (Supplementary Table 3).

**Table 1:** Oral cholera vaccine (OCV) coverage by strata among participants in the coverage survey, Goma, North Kivu, Democratic Republic of the Congo, 2022 (N = 3,258 individuals, n = 599 households).

**Stratum**	**N individuals (n households)**	**Number of doses targeted**	**1 dose (95 % CI)**	**2 doses (95 % CI)**	**At least 1 dose (95 % CI)**
All strata	3,258 (599)	0, 1, or 2	33.2 (28.8–37.9)	11.8 (8.9–15.3)	46.4 (41.8–51.0)
Strata targeted for at least one dose		1 or 2	35.9 (31.1–41.1)	13.5 (10.2–17.5)	50.1 (45.1–55.0)
1	373 (63)	2	38.3 (31.7–41.3)	11.5 (3.8–33.3)	50.7 (40.1–61.2)
2	470 (80)	2	31.8 (22.9–42.4)	11.5 (5.9–21.3)	43.3 (31.2–56.3)
3	426 (86)	2	33.4 (20.6–49.3)	18.7 (10.5–31.2)	55.8 (44.9–66.2)
4	519 (88)	0 (never targeted)	22.8 (16.1–31.2)	3.4 (1.2–9.4)	26.3 (19.2–34.9)
5	425 (71)	2	43.4 (32.1–55.3)	13.8 (6.9–25.7)	57.3 (44.3–69.3)
6	406 (76)	2	27.7 (14.4–46.6)	18.5 (8.5–35.7)	46.3 (31.1–62.1)
7	386 (87)	2	35.9 (25.3–48.2)	6.8 (2.6–16.7)	42.8 (30.6–55.9)
8	253 (48)	1	43.8 (22.5–67.7)	10.5 (4.4–23.2)	55.4 (30.8–77.5)

### Socio-demographic factors

Coverage with at least one dose in the 2019-vaccinated zone was 52.4 % (95 %CI: 46.1–58.6), which was not significantly different from that in the 2020-vaccinated zone (47.5 %, 95 %CI: 39.8–55.3). Men’s vaccination coverage was significantly lower than women’s: 43 % of men reported vaccination versus 57 % of women (OR: 0.71, 95 %CI: 0.62–0.81). Compared with participants over 15 years old, those aged 5–14 were more likely to have received at least one dose (OR: 1.6, 95 %CI: 1.3–1.9). Those below 5 years were significantly less likely to have received at least one dose (OR: 0.28, 95 %CI: 0.21–0.37) (Table 2).

**Table 2:** *Oral* Cholera *Vaccine Coverage and covariates, Goma, Democratic Republic of the Congo, August 2022.* Percentages are calculated by column.

	Overall** n (%), n = 3258	**Vaccinated with at least 1 dose** n (%), n = 1492	**Unvaccinated** n (%) n = 1766	**Odds Ratio** (95 %CI)
**Sex**				
Female	1 693 (52.0)	844 (56.6)	849 (48.1)	ref
Male	1 541 (47.3)	640 (42.9)	901 (51.0)	0.71 (0.62–0.81)
Unknown sex^2^	24 (0.7)	8 (0.5)	16 (0.9)	−

**Age**				
1–4 years	293 (9.0)	55 (3.7)	238 (13.5)	0.28 (0.21–0.37)
5–14 years	854 (26.2)	483 (32.4)	371 (21.0)	1.6 (1.3–1.9)
15 years and over	2 069 (63.5)	934 (62.6)	1135 (64.3)	ref
Unknown Age^2^	42 (1.3)	20 (1.3)	22 (1.2)	−

History of moving**^,^^1^				
Remained in same health area	2746 (84.3)	1324 (88.7)	1422 (80.5)	ref
Moved from other health area of Goma city	275 (8.4)	118 (7.9)	157 (8.9)	0.84 (0.65–1.1)
Moved from other health area outside of Goma city	227 (7.0)	41 (2.8)	186 (10.5)	0.23 (0.16–0.32)
Moved from any health area (within or outside Goma city combined)	502 (15.4)	159 (10.7)	343 (19.4)	0.50 (0.40–0.61)
Unknown moving status	10 (0.3)	9 (0.6)	1 (0.1)	−

History of severe diarrhea**^,^^1^				
No	3058 (93.9)	1413 (94.7)	1645 (93.1)	ref
Yes	173 (5.3)	63 (4.2)	110 (6.3)	0.68 (0.48–0.93)
Unknown history^2^	27 (0.8)	16 (1.1)	11 (0.6)	−

** *Since January 2019*.

1 *OR adjusted for age and sex.*

2 *Participants with unknown values are excluded from Odds Ratio calculations*.

### Population movements

Fifteen percent of participants reported moving from a different HA since 2019 (from 6.4 % in stratum 2 to 32.9 % in stratum 1), of which 45 % (7 % of total) had arrived from outside Goma city. Among those who had moved since January 2019, 31.7 % were vaccinated with at least one dose, which is significantly lower than the proportion vaccinated among people with no history of moving (48.2 %). People who had moved from a HA outside Goma had a lower probability of being vaccinated, with only 18.1 % (95 %CI: 13.6–23.5) of those moving from a HA outside of Goma being vaccinated with at least one dose, compared to 47.9 % (95 %CI: 46.1–49.6) of those coming from a Goma HA.

### Episodes of severe diarrhea

Among participants, 5.4 % reported to have experienced severe diarrhea, of which 19.3 % reported a cholera diagnosis during this diarrhea episode. The proportion of participants with a diarrhea history ranged from 2.2 % (95 %CI: 1.1–4.1) in stratum 6 to 10.0 % in stratum 1 (95 %CI: 7.4–13.6) and 13.3 % in stratum 8 (95 %CI: 9.6–18.1).

## Discussion

A key finding of the study relates to population movements being an important factor influencing Goma’s OCV coverage. People’s permanent relocation in and out of Goma accelerates coverage waning, with some vaccinated persons leaving and unvaccinated ones arriving. Moreover, an important number of doses intended for targeted areas were received by people living in non-targeted areas, suggesting daily movement across HA during campaigns for work or leisure activities probably affected coverage levels. This coverage dilution underscores the importance of fully vaccinating geographically contiguous areas (e.g. entire health zones) during preventative OCV campaigns, especially in big cities. This result was considered in designing future national vaccination strategies, through the recent development of the 2023–2027 Multi-Sectoral Plan for Cholera Elimination.

Other studies have shown that targeted OCV campaigns in African urban settings can reach about 80 % 1-dose initial coverage, [14], [17], [18] or even higher in Bangladesh [19]. Despite initial rapid assessments yielding high coverage estimates in vaccinated areas, the exact initial overall coverage after Goma campaigns remains unknown. The 2022-measured coverage of at least one dose (46.4 %) is lower than expected with over half of the population unvaccinated, leaving people vulnerable to cholera and far from reaching sufficient coverage levels for herd immunity.

The low 2-dose coverage of 13.5 % in targeted areas also suggests that people vaccinated were different in both rounds of the campaigns. This could relate to frequent population movements, though the 2020 2nd dose campaign happening during Covid-19 pandemic times, or recall bias, could also have played a role.

Men were less likely to be vaccinated than women. A similar trend was observed in other OCV coverage surveys in similar contexts, and could be related to men more often working away from home and thus absent when vaccinators pass door-to-door [17]. The campaign-to-survey time explains the lower coverage in children under 5, part of them being born post-campaigns. Older children, on the other hand, were more likely to be vaccinated than adults; as vaccines are rarely proposed to adults through mass campaigns; this could reflect a misunderstanding on the vaccine target, or lower acceptance in adults.

Operationally, all these elements need to be considered in planning mass campaigns, particularly population movements. For instance, vaccination strategies may be adapted to population’s schedule, especially adult men, notably by reinforcing vaccination at work sites, shops, or streets in addition to visiting residences door-to-door. Modification of vaccination hours, or neighborhood-level community strategies could also be considered. In addition to periodic mass campaigns, routine campaigns to vaccinate new arrivals in migratory areas, younger children and previously missed persons could be beneficial; however, such strategies require large and regular amounts of available OCV [9].

Campaigns would also benefit from more awareness programs focused on adults’ vaccine uptake, and education on vaccine schedule’s importance. While delay between Goma campaigns reached 5–6 months both years, shorter delays may also facilitate recall and awareness about 2nd dose. In addition, systematic assessment of the post-campaign coverage using WHO-recommended cluster methodology could also help evaluate the campaigns. Finally, using OCV in combination with other complimentary multi-sectoral interventions such as improvements to hygiene and water supply systems, could help maximize results [20]. Further studies will be needed to investigate the impact of such mass OCV campaigns in urban endemic contexts, and qualitative investigation could also help develop additional strategies and increase coverage in such contexts.

People living along the periphery of the city (strata 1 and 8) had higher self-report of diarrhea. These areas are known to welcome more new arrivals, especially displaced people. Those with a history of diarrhea were significantly less likely to have been vaccinated, which could suggest some protective effect of the vaccine. Participants were more likely to recall the episode only if a cholera diagnosis was made, potentially influencing these results. Moreover, other factors including socioeconomic status or access to protective measures could be influencing this effect, meriting further investigation.

One key limitation of this study is recall bias, as participants may have forgotten if or when they or their family members were vaccinated (and how many doses), and if they had diarrhea. This was mitigated by describing the cholera vaccination in detail during interviews, and the fact that OCV is the only oral vaccine also targeting adults, which may have facilitated recall. Given that this study only included people living in Goma at the time of survey, we were unable to study population movements from within Goma to outside of the city, in particular people who were vaccinated but subsequently left.

## Conclusion

These results give unique insights on OCV coverage in targeted urban settings after an elapse of time, and illustrate that targeting non-contiguous zones for vaccination in urban contexts with frequent population movements can result in diluted and suboptimal final coverage levels. Thus, campaigns targeting larger geographic areas and entire population groups could result in higher vaccination coverage in such contexts. Results also suggest the importance of adapted vaccinations strategies, including adapting to adults’ schedule, planning for catch-up campaigns, strengthening awareness programs or considering integrated combined approaches for cholera.

## Authors’ contributions

The following authors (alphabetical by last name) were part of the Epicentre-MSF DRC cholera Working Group and made valuable contributions to investigation, data curation and interpretation of the results: Isabel Amoros-Quiles, Richard Safari Balegamire, Flavio Finger, Patient Kamavu, Joseph Kasereka, Christopher Mambula, Germain Mweha, Johanne Sekkenes, Abdoul Kassim Toure.

## Funding

This work was supported by the Wellcome Trust grant number: [215689/Z/19/Z], co-financed by Wellcome Trust and Foreign Commonwealth Development Office (FCDO). The funder was not directly involved in the data collection, analysis, or decision to publish these results.

## CRediT authorship contribution statement

**Emily Briskin:** Writing – original draft, Visualization, Methodology, Investigation, Formal analysis, Data curation, Conceptualization. **Stéphane Hans Bateyi Mustafa:** Writing – review & editing, Conceptualization. **Rachel Mahamba:** Writing – review & editing, Methodology, Investigation, Data curation. **Deka Kabunga:** Writing – review & editing, Conceptualization. **Janvier Kubuya:** Writing – review & editing, Conceptualization. **Klaudia Porten:** Writing – review & editing, Supervision, Conceptualization. **Laurent Akilimali:** Writing – review & editing, Supervision, Conceptualization. **Placide Okitayemba Welo:** Writing – review & editing, Supervision, Conceptualization. **Anaïs Broban:** Writing – review & editing, Writing – original draft, Visualization, Supervision, Project administration, Methodology, Conceptualization.

## Declaration of competing interest

The authors declare the following financial interests/personal relationships which may be considered as potential competing interests: Anais Broban reports financial support was provided by Wellcome Trust. Anais Broban reports a relationship with Epicentre that includes: employment. Emily Briskin reports a relationship with Epicentre that includes: employment. Rachel Mahamba reports a relationship with Epicentre that includes: employment. Klaudia Porten reports a relationship with Epicentre that includes: employment. Placide Okitayemba Welo reports a relationship with Ministry of Health DR Congo that includes: employment. Laurent Akilimali reports a relationship with Ministry of Health DR Congo that includes: employment. Deka Kabunga reports a relationship with Ministry of Health DR Congo that includes: employment. Janvier Kubuya reports a relationship with Ministry of Health DR Congo that includes: employment. Stephane Hans Bateyi Mustafa reports a relationship with Ministry of Health DR Congo that includes: employment. The authors have no other conflict of interest to declare. If there are other authors, they declare that they have no known competing financial interests or personal relationships that could have appeared to influence the work reported in this paper.

## Data Availability

Data are publicly available under the following doi: 10.5281/zenodo.12651251.
