# Supplementary material for: Oral cholera vaccine coverage in Goma, Democratic Republic of the Congo, 2022, following 2019–2020 targeted preventative mass campaigns
Source: Vaccine X. 2024 Sep 12;20:100555. doi: 10.1016/j.jvacx.2024.100555 (PMC11417590; doi:10.1016/j.jvacx.2024.100555)
Supplement: Supplementary Data 1 [file mmc1.docx]

**Supplementary Data: Extracts of survey protocol, based on MSF template protocol for coverage surveys**

Protocol title: Vaccination coverage survey for oral cholera vaccination in Goma,

Democratic Republic of the Congo

1. Introduction [extract]

## CHOLERA PREVENTION AND RESPONSE IN GOMA

OCV vaccination in Goma city has occurred in 2 distinct rounds: one in 2019 and another in 2020. Due to the number of available vaccines at the time of the campaign and geographical disparities of cholera suspect cases notifications within the city, not all health area were targeted for vaccination. In 2019, a first round of vaccination targeted 13 health areas of the city, with first dose distributed in May and second in October. In 2020, an additional 10 health areas were targeted for vaccination in January and June. In addition, one health area was targeted for 1 dose only in 2020. High administrative coverages were reached in each of the 4 campaigns, confirmed by a rapid monitoring assessment but no full vaccine coverage using WHO methodology. Maps and administrative coverage estimates of the campaigns are shown in the figure below.


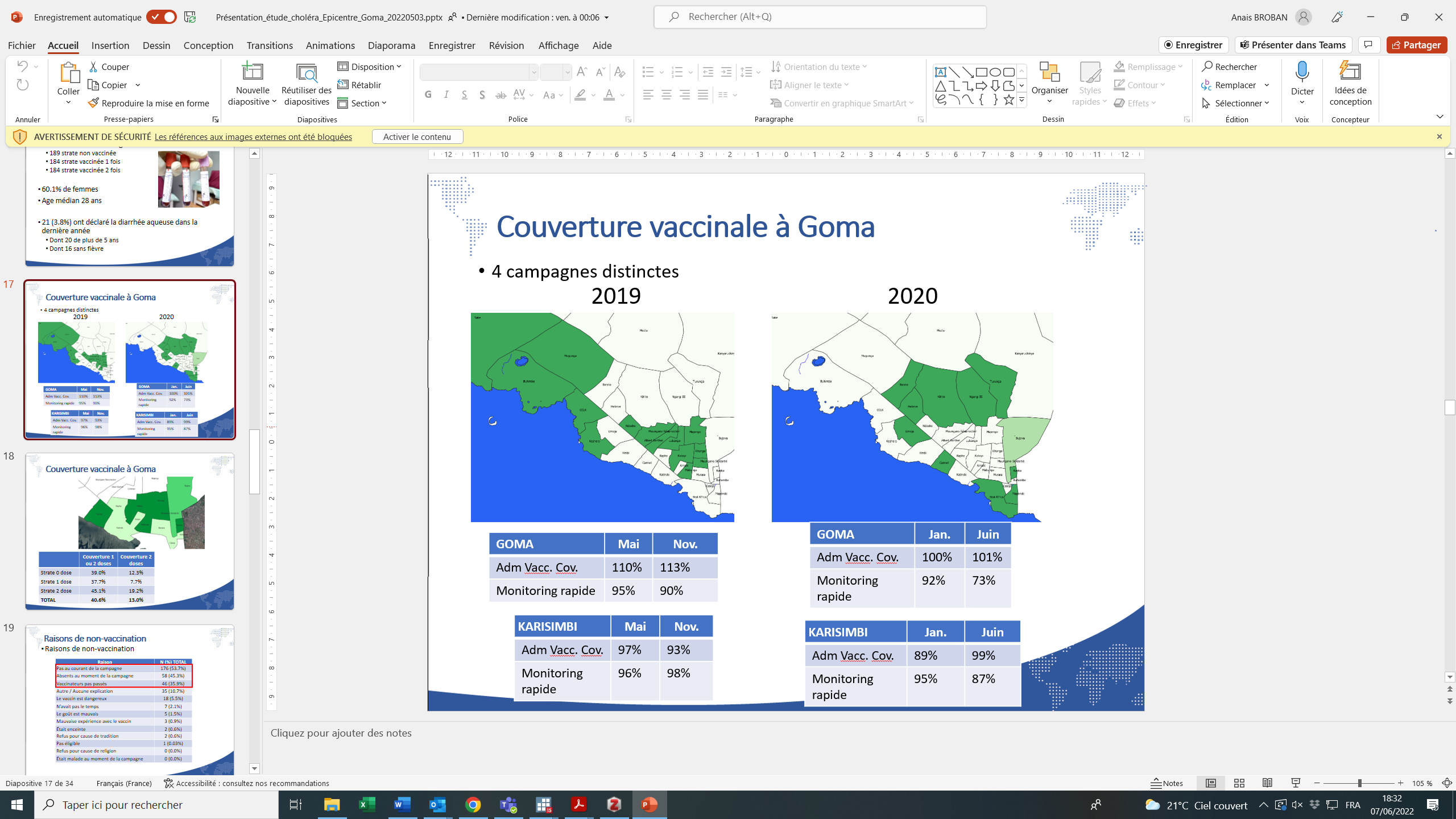


Figure 4 Targeted health area in OCV vaccination campaigns, 2019 and 2020, and results of administrative coverage and rapid monitoring assessment in Goma and Karisimbi health zones

3. Objectives

## 3.1. Primary objectives

- To describe the current overall vaccination coverage for persons aged ≥1 year with one, two, or three or more more doses of OCV
- To describe the strata-specific vaccination coverage for persons ≥1 year for single-dose and two-dose OCV (there are 8 strata which differ by geography and whether they were targeted for OCV single dose or two doses)

## 3.2. Secondary objectives

- To describe coverage of OCV doses (1, 2, or 3 or more) received by different age groups (less than 5 years old, 5-15 years old and over 15 years old)
- To describe the reasons for non-vaccination during the different vaccination opportunities, and quantitatively evaluate their relative importance.
- To estimate level of population movement and how this may impact evolution of vaccination coverage over time
- To provide recommendations for vaccination strategies, surveillance, and implications for ongoing research in Goma

4. Survey Design [EXTRACT]

The vaccination coverage survey will be conducted using two-stage cluster sampling, as an adaptation of the standardized method recommended by the World Health Organization (WHO)^[[1]](#footnote-2)^ [this methodology is described in Chapter 6.2]. This method is chosen as a reliable list of households and their placement across the geographical area is not available for Goma.

Determination of vaccine status will be done by, in order of preference, (a) examination of individual OCV-specific vaccination cards which record OCV doses received for eligible persons ≥1 years of age and (b) verbal self-reporting of vaccination status.

In each household, a single respondent ≥18 years of age (or emancipated minor <18 years) will be asked about the vaccination status for each household member. Given that the mass oral cholera vaccination campaigns were held in 2019 and 2020, prompts for the dates of the campaigns (e.g., salient or memorable holidays, harvest times, conflict events, etc.) will be used to improve recall around those vaccination events. To improve recall regarding oral cholera vaccination that may have been distributed as part of a CATI response, the CATI response methods and accompanying distribution of hygiene kits will be described. For the self-reporting of vaccination status, the respondent will be asked if the household member ever received a vaccination or medication by mouth/orally in the last years. To distinguish from oral polio vaccine, OCV was the only oral vaccine distributed to adults in the last years in Goma. History of cholera disease during the lookback period will also be recorded, as this can help indicate the immunity level of the population.

5. Target population [EXTRACT]

All persons ≥1 year living in Goma during the time of the survey will be candidates to be included.

## 5.1. Inclusion and exclusion criteria

Persons will be included in the survey if they satisfy all the following criteria:

- Living in the randomly selected household (see chapter 5.1. for the definition of a household)

and

- Informed consent has been given by the persons themselves or their parents/guardians/caretakers (see chapter 9.1. for details on the informed consent)

Persons will be excluded from the survey if they satisfy one of the following criteria:

- Refusal to participate in the survey (persons themselves or their parent/guardian/caretaker)

*or*

- Inability to locate a household representative for the selected household after two attempts to trace him/her

6. Definitions

## 6.1. Household Definitions

*Definition of household*

A household will be defined as a group of people who are under the responsibility of the same head of household and slept on the same parcel the previous night. All members of the household ≥1 year of age will be included, no matter the relation with the other members.

*Definition of head of household*

The head of household is defined as follows:

- Adult household member ≥18 years of age, (or an emancipated minor between age 16 and 18) *and*
- Can give accurate information on all demographic issues in his/her
  household and
- Is present at the time of the survey

A household will be excluded from the survey if none of the household members fulfil all these criteria.

## 6.2. Vaccination definitions

- OCV (single dose) vaccinated by card
  - An individual who received one dose of OCV over the lookback period (from January 2019 until the time of the survey). This is confirmed by the presentation of a vaccination card where receipt is clearly marked for a single dose.
- OCV (single dose) vaccinated by verbal confirmation
  - An individual who received one dose of OCV over the lookback period. This is confirmed on interview by verbal history of the participant or his/her parents/guardians/caretakers, but without verification using a vaccination card.
- OCV (two doses) vaccinated by card
  - An individual who received the first and second doses of OCV over the lookback period. This is confirmed by the presentation of a vaccination card where receipt is clearly marked for both the first and second doses.
- OCV (two doses) vaccinated by verbal confirmation
  - An individual who received the first and second dose of OCV over the lookback period. This is confirmed on interview by verbal history of the participant or his/her parents/guardians/caretakers, but without verification using a vaccination card.
- OCV (two doses) vaccinated by card and verbal confirmation (mixed verification)
  - An individual who received the first and second doses of OCV over the lookback period. This is confirmed by the presentation of a vaccination card where receipt is clearly marked for either the first or second dose and, the other dose is confirmed on interview by verbal history of the participant or his/her parents/guardians/caretakers.
- OCV (three or more doses) vaccinated by card
  - An individual who received three or more doses of OCV during the lookback period. This is confirmed by the presentation of a vaccination card(s) where receipt is clearly marked for at least 3 doses.
- OCV (three or more doses) vaccinated by verbal confirmation
  - An individual who received at least three doses of OCV within the lookback period. This is confirmed on interview by verbal history of the participant or his/her parents/guardians/caretakers, but without verification using a vaccination card.
- OCV (three or more doses) vaccinated by card and verbal confirmation (mixed verification)
  - An individual who received the at least three doses of OCV within the lookback period. This is confirmed by the presentation of a vaccination card where receipt is clearly marked for either the first, second, or third/additional dose and, the other dose is confirmed on interview by verbal history of the participant or his/her parents/guardians/caretakers.
- OCV-Not vaccinated
  - An individual who had no written vaccination record and does not recall being vaccinated with any dose. This is confirmed on interview by the participant or his/her parents/guardians/caretakers stating that no OCV was received.
- OCV-Unknown
  - An individual or his/her parents/guardians/caretakers do not recall if the survey participant was vaccinated
- Total Number of doses
  - The total number of doses received will be the sum of all doses reported by card and self-reported using verbal confirmation, during the lookback period
  - For those who self-report as being vaccinated but with an unknown number of doses, and no card is available, the interviewer will work with the participant to record the best estimate.

7. Sample size and sampling

## 7.1. Sample size Calculation

The sample size estimate is based on:

- Expected vaccination coverage ratios: we assume 50% prevalence for two-dose coverage of OCV among persons ≥1 year. 50% produces the largest possible sample size and is appropriate since the overall coverage is highly uncertain. A previous serosurvey in Goma in January 2022 found a prevalence estimate of 40,6 % (95%CI 36.5-44.8).
- Desired precision of the results: 8 to 10% precision at strata level*.*
- Design (cluster) effect*:* design effect of 2 (high hetereogeneity between clusters is assumed)
- Non-response: assumed to be 20% (high, due to absence, refusal, etc.)

For the sample size calculation, an expected vaccination coverage of 50%, an alpha error of 0.05 (confidence level of 95%), a precision of 8% to 10% and a design effect of 2 and a non-response of 20% will be assumed. Examples of sample size calculations with different precision levels and design effects can be found in Table 1.

*Table 1 : Sample size calculations for OCV coverage studies*

| **Criteria** | **Est 1** | **Est 2** | **Est 3** |
| --- | --- | --- | --- |
| Expected vaccination coverage | 50% | 50% | 50% |
| Confidence level | 95% | 95% | 95% |
| Design effect | 2 | 2 | 2 |
| Precision | **+ / - 5%** | **+ / - 8%** | **+ / - 10%** |
| Non-response | 20% | 20% | 20% |
| Mean household size | 5.2 | 5.2 | 5.2 |
| Mean household size ≥1 year | 5.0 | 5.0 | 5.0 |
| Nr. persons ≥1 year to be sampled | **922** | **362** | **232** |
| Nr. households to be sampled | **185** | **73** | **47** |
| Cluster designs (per strata) | **20 x 10** | **20 x 4** | **20 x 3** |

Sample size was calculated with the help of “OpenEpiMenu*"*^[[2]](#footnote-3)^*.* Based on a reported average household size of 5.2 (2018 MICS) with 96% of the population aged ≥1 year [14], we can expect on average a frequency of 5.0 (e.g. 5.2*0.96) eligible persons ≥1 year per household.

For Est 2, to include 362 persons ≥1 year of age, 73 households [362 divided by 5.0 = 73] households need to be included in the survey, thus 20 clusters of 4 households. Note that 15 is recommended as the minimum number of clusters in a vaccination coverage survey to capture heterogeneity [15].

Given that the survey will be carried out in 8 strata, the total households to be sampled is 640 [20 * 4 * 8] and total persons ≥1 year of age is about 3 200 [20 individuals*20 clusters*8] (for Est 2).

## 7.2. Sampling procedure

A two-stage cluster sampling methodology will be chosen as an adaptation of the standardized method recommended by the WHO.

By strata, in the first stage, 20 clusters will be selected from the sampling frame of all geographic strata. Cluster allocation will be by simple geographic random sampling. Using delimitation boundaries file, 20 GPS points will be randomly chosen in each strata using geographic mapping software*.*

**An adapted version of the 2005 standard WHO/EPI methodology** will be used to select 4 households within each cluster. The first house to be selected will be the closest to the randomly selected spatial point. If two dwellings are equidistant, the left-hand dwelling will be selected. If no dwelling is available for inclusion within about 50 meters, that GPS point will be excluded. In case several households are sheltered in the same selected dwelling, one of them will be randomly selected by surveyors upon arrival. After this first household has been included, the survey team will pick the next households using the following systematic procedure based on proximity:

• stand in the front door of the previous household and look outside

• walk to the left, crossing 5 households (and staying within the neighbourhood boundaries as defined by community leaders)

• the 5th household is to be selected for the next interview

• One household every 5 households will be interviewed with this technique, until reaching a total of 4 interviewed households.

For all household surveyed, if the head of the household (or his or her representative) is absent, the investigators will ask when he or she will return so that they can come back later that day to survey the household. If the head of the household is not expected to return that day, then the investigators will interview the next household within the dwelling, or in the next closest dwelling if there are no other households available to interview.

As the survey will happen in a city, it is not expected that all households of a selected cluster are included in the study before completing the required number of households.

If for unforeseen reasons a selected neighborhood (cluster) cannot be visited, it will be replaced by selecting a new random GPS point in the strata.

Any areas excluded from the sampling frames for reasons of insecurity or inaccessibility for some other reason (e.g limited access due to poor roads or weather) will be documented in the report, as will any unexpected exclusions resulting in replacement of a cluster.

8. Data collection

The field supervisor will inform the head of neighborhood selected (=clusters) according to the sampling (see chapter 6.2.) 1-2 days the survey teams will visit them.

The purpose of the survey will be explained by interviewer team to heads of the neighborhoods on the day of the survey before conducting interviews in their neighborhoods. Furthermore, it will be clearly explained to the heads of the neighborhoods that they are freely allowed to decline the participation of their neighborhoods without any consequences or penalty. In this case the neighborhoods will be replaced by selecting another random GPS point within the strata.

In the households randomly selected according to the above methodology, the interviewer team will explain the purpose of the survey to the head of the household/survey participant or the parents/guardians/caretakers in the language he or she is familiar with and verbal consent obtained to conduct the interviews and documented on the questionnaire. All refusals will be recorded and those forms retained to document participation rate.

All persons over 1 year old in the identified households in the target population are included in the survey, including in the final household of a cluster, even if this exceeds the total target of persons for the cluster.

A standardized pre-piloted questionnaire will be used to collect the following data for each participant of the cohort at recruitment:

- Demographic data: age, sex, number of people in the household
- Vaccination status: verbal and card confirmation
- History of cholera during the lookback period
- Reasons for non-vaccination
- Vaccination source (mass campaign, CATI, or other)
- Approximate arrival date of the participant in their current dwelling/ health area

9. Data entry and AnalysIS [EXTRACT]

No name-related data will be collected during the survey, reducing the risk that participants will be identifiable after the survey has been completed. An electronic database will be generated from the questionnaires and this database will be password protected. Any paper versions of the questionnaires (paper versions) and the electronic database will be stored at the MSF Headquarters or country management level for 5 years after the survey. Access to the electronic and paper version of the survey will be restricted to the co-investigators of the study and the Medical Coordinator. After 5 years the paper copies of all the questionnaires will be destroyed.

10. Ethical Principles [EXTRACT]

## 10.1. Verbal consent form and confidentiality

Verbal consent will be sought from every household, with the designated head of household answering the questionnaire for all relevant members of the household (see Annex). He/she may choose to delegate answering the questionnaire to another member of the household, or to individuals regarding their own vaccination status if relevant.

All participants included in the survey will have the survey activity explained to them in a language with which they are familiar, in this case their choice of French or Swahili. Everyone will be offered the opportunity to refuse participation in the survey at any time without penalty and no incentives or inducements will be provided to any respondents. Everyone approached for the survey is completely free to participate or not.

Privacy and confidentiality in the data collected from the participants will be ensured both during and after the conduct of the survey. Participant names will not be recorded on questionnaires, and individual person records will be linked only to a household number throughout the data entry and analysis process. Any data that could be combined with other data sources to make individual records potentially identifiable will not be distributed outside the survey location, or appear in any report or publication.

**Appendix 2:**  **Informed Consent sheet and verbal form, head of household**

**Introduction and goals of the study:**

Thank you for taking the time to listen to our information about this study, my name is.........................................................................

As you might already know Epicentre/Médecins sans Frontières (MSF) jointly with and the Ministry of Health are supporting health care projects and research in this area.

Today we are carrying out a survey to estimate the numbers of individuals that have been vaccinated against cholera. With this survey, Epicentre/MSF hopes to improve their work as a medical organisation in the use of vaccination to protect the community against outbreaks of cholera. Secondly, MSF/Epicentre’s intention is to use the results of the survey to raise awareness of the vaccination situation in your community, to support the MoH to improve future health activities. The name of your neighborhood may be used in or reports, but details of individual households will not be disclosed. The data that we collect could be shared with health authorities.

**Why was my household chosen for this survey?**

Your household was chosen because you are living in the area known to be endemic for cholera (Goma) that was selected for participation in this study. As you match these criteria, we would like to propose to you to participate.

**What does it mean for me and my family to participate?**

If you agree to participate to the survey, we will ask you questions such as the vaccination history of the members of your household and yourself. We will also ask general questions regarding how many persons are living in your household. This interview will be conducted by trained staff. The interview should not last more than 10 minutes.

**Do I have to take part in the survey?**

Your participation in this survey is voluntary and you are free to participate or not, even if the head of the neighbourhood/village has agreed. If you do not wish to participate, it will not affect the usual medical care you or your family will receive in the health centres, now or in the future. If you choose to participate, you may decline to answer any question without any consequences and you may also decide to stop your participation at any time during the study without giving reason. In this case, we will stop the procedures with you, and we will ask you if you accept that we keep the data collected so far or not.

There will be no compensation for your participation in this survey. We will not distribute anything.

**What are the possible benefits?**

There is no direct benefit for you but the information that we collect in this study will contribute to a better understanding of cholera cases and transmission, and of the impact of cholera vaccination campaigns. This information will thus help us to protect you and your community better from cholera in the future.

**What are the possible risks?**

We do not expect any physical risk for you or your family members to participate in this survey. However, asking you personal questions about your health or giving out personal information may be upsetting for you. You can refuse to answer any questions without any consequences. You are also free to stop the interview at any time without any further impact.

There is also a risk related to the loss of confidentiality. The interviewers will be trained to ensure that your privacy is respected and to ensure the confidentiality of the discussion. To decrease this risk, they will be meticulously trained in interview techniques and will keep study related documents secured in a locked cabinet accessible by the study team only. The data we collected will not contain the names of the participants.

**How will our personal information be protected?**

The information you provide will remain confidential. We will not record your name or the names of the members of your household. Only the names of the participating neighbourhoods/villages will be recorded. A survey number will be assigned to each household, without identifying the participants and we will not record the location of your house. Only the study team can access the data we collected. The data will be collected on tablets (or paper and then entered in a computer). The paper study documents will be kept in a safe locked cabinet either in Goma, Kinshasa, or in Europe (at MSF’s headquarters) and will be only accessible by the study team. The electronic data will be stored on a secured encrypted server at Epicentre in France. All data and study documents will be archived for 5 years and then destroyed.

**Will the results of the survey be shared?**

Once the study is completed, the information collected will be analysed and the health local authorities that participated in the survey will receive a summary of the results.

The final report will be shared with all partners (Ministry of Health, MSF-OCP, Epicentre and Wellcome Trust). We will also work with the local authorities to share the results of the study with the community.

The results of the survey may also be used in international publications.

**Who has reviewed the study?**

The Ethical Review Board of the Ministry of Health of DRC *insert the name of the local ethics committee and its address* on [20/07/2022] and the Ethics Review Board of MSF on the [11/08/2022] have reviewed and approved the study.

**Who can I contact if I have a complaint or question?**

Complaints or questions should be addressed to either:

- Name Surname, local study coordinator, MSF OCP or Epicentre, DRC at provide an active local phone number [xxxxxxxxx@paris.msf.org](mailto:xxxxxxxxx@paris.msf.org)(contact local)
- Name Surname, epidemiologist, Epicentre at [xxxxxxx@epicentre.msf.org](mailto:xxxxxxx@epicentre.msf.org) (principal investigator)

Do you have any questions about the survey? Do not hesitate to ask us about our work, we will be happy to answer.

[After answering the questions] We thank you very sincerely for your collaboration and contribution.

In case you have any questions after the interview has been completed, you are free to contact the survey supervisor at the contact above.

***Please go through the information sheet before seeking consent***

The participant has understood the information sheet and his/her questions have been answered to his/her satisfaction. He/she gives voluntary consent to answer the questions in the questionnaire for the household.

The participant understands that he/she is free to withdraw from the study at any time.

**Does the head of Household agree for their household to participate in this study?**


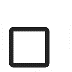
 YES
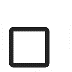
 NO

Aire de Santé name: *____________________________*

Household number: *______*

*I have explained the purpose of this research to the participant. To the best my knowledge, he/she understands the purpose, procedures, risks and benefits of this research.*

Date: ___ ___ / ___ ___ / 2022 (day/month/year)

Data collection team number : ____

Name of the person taking the consent: _________________________________

Signature of the person taking the consent: _________________________________

*[Only if the participant provides informed verbal consent may the household be included.]*

[The interviewer can only proceed with data collection if the head of household gives informed verbal consent].

Do not forget to leave an information sheet with the participant

**Supplementary Table 1: Strata definitions and descriptions, Oral Cholera Vaccine Coverage Survey, Goma 2022**

| **Strata nb** | **Health areas included** | **Number of OCV doses received in 2019-2020 campaigns** | **Population of health areas*** | **Population density (inhabitant/km^2^)**** | **Urban, Suburban, or Rural** | **Contained displaced persons camps** | **Bordering Lake** |
| --- | --- | --- | --- | --- | --- | --- | --- |
| 1 | Mugunga, Buhimba | 2 | 63363 | 3278 | Suburban and rural | Yes | Yes |
| 2 | CCLK, Keshero, Umoja | 2 | 126664 | 7934 | Urban and suburban | No | Yes |
| 3 | Baraka, Hebron, Nodsho, Kiziba, Ngangi III | 2 | 179432 | 10215 | Urban and suburban | No | No |
| 4 | Himbi, Rapha, Katindo, Amani, Murara, Casop, Kahembe | 0 | 149145 | 12517 | Urban | No | Yes |
| 5 | Muungano Résurrection, Albert Barthel, Lubango, Carmel | 2 | 146592 | 15535 | Urban | No | Yes |
| 6 | Majengo, Virunga, Katoyi, Méthodiste | 2 | 163793 | 22645 | Urban | No | No |
| 7 | Heal Africa, Mapendo, Kasika, Mabanga, Muungano Solidarité | 2 | 125655 | 11557 | Urban | No | Yes |
| 8 | Bujovu | 1 | 39735 | 7046 | Suburban | Yes | No |

*Source: Bureau central de zone de Goma, Karisimbi et Nyiragongo. The numbers may not include population of the displaced persons camps set up on those areas.

**Calculated using population data and health area mapping in QGIS software

**Supplementary Table 2: Questionnaire (implemented with Kobo Collect), Oral Cholera Vaccine Coverage Survey, Goma 2022. All persons over 1 year old.**

| **Variable name** | **Label** | **Note** | **Options** | **Branching** |
| --- | --- | --- | --- | --- |
| inc_date | Date d'inclusion |  | ___/___/______ |  |
| equipe | Choisir le numéro d'équipe des enquêteurs |  | ____ |  |
| grappe | Donner le numéro de la grappe | (le titre du point GPS) | __________ |  |
| menage | Choisir le numéro du ménage dans la grappe | (enquêter 4 ménages par grappe au maximum) | ____ |  |
| cons | Le chef du ménage a-t-il reçu les informations nécessaires et donné son consentement verbal ? |  | oui, Oui  non, Non |  |
| mm | Combien de membres dans ce ménage sont âgés d'au moins 1 an ? |  | ____ | ${cons} = 'oui' |
| note1 | Répondre aux questions suivantes pour chaque membre du ménage qui est âgé d'au moins 1 an. |  |  | ${cons} = 'oui' |
| sexe | Cette personne a quel sexe? |  | M, Masculin  F, Féminin  U, Inconnu |  |
| age | Cette personne a quel âge (en années)? |  | ____ |  |
| doses | Depuis janvier 2019, vous avez reçu combien de doses du vaccin contre le choléra? |  | 0, 0  1, 1  2, 2  3plus, 3 ou plus  U, Inconnu |  |
| dose1_vax | Pour la première dose d'OCV reçue depuis janvier 2019, cette personne est: |  | V, Vacciné, confirmation verbale  C, Vacciné, confirmé avec la carte  U, Inconnu | ${doses} != '0' and ${doses} != 'U' |
| photo_carte | Prenez une photo de la carte de vaccination | Si plusieurs cartes de vaccination OCV, tirer une seule photo qui montre tous les cartes côté à côté. |  | ${dose1_vax} = 'C' |
| dose1_date | Donner la date approximative de la première dose. | Si le jour est inconnu, sélectionner le 15 du mois. Si le mois est inconnu, sélectionner le mois de juin. | ___/___/______ | ${doses} != '0' |
| dose1_dist | Pour la première dose, préciser le type de distribution |  | M, Campagne de masse  C, CATI  X, Autre  U, Inconnu | ${doses} != '0' and ${doses} != 'U' |
| dose1_raison_nonvax | Pour la première dose, raison de la non-vaccination |  | 1, Pas au courant de la campagne  2, Les vaccinateurs ne sont pas passés à la parcelle  3, Etait absent lorsque les vaccinateurs sont passés  4, Etait âgé moins d'un an  5, Etait enceinte  6, Refus pour cause de tradition  7, Refus pour cause de religion  8, La vaccination est dangereuse  9, Mauvaises expériences avec d'autres vaccins dans le passé  10, Il n'y avait pas assez de vaccin  11, N'a pas eu le temps  12, Etait malade (à la maison)  13, Etait hospitalisé  14, Le goût est mauvais  15, N'habitait pas dans une zone ciblée  16, Aucune explication | ${doses} = '0' |
| zds | La première dose a été reçue dans quelle Zone de Santé ? |  | Goma, Goma  Karisimbi, Karisimbi  Kirotshe, Kirotshe  Nyiragongo, Nyiragongo  hors goma, Autre (hors Goma) | ${doses} != '0' and ${doses} != 'U' |
| ads | Pour la première dose, préciser là où la dose a été reçue | A Goma, choisir l'aire de santé. Hors Goma, choisir la province. | Liste des aires de santé de la ville de Goma | ${doses} != '0' and ${doses} != 'U' |
| agglom | Si la première dose a été reçu hors Goma, donner le nom de la Zone de santé ou l'agglomération la plus proche de la site où la vaccination a été reçu. |  | ________________ | ${zds} = 'hors goma' |
| dose2_vax | Pour la deuxième dose d'OCV reçue depuis janvier 2019, cette personne est: |  | V, Vacciné, confirmation verbale  C, Vacciné, confirmé avec la carte  U, Inconnu | ${doses} = '2' or ${doses} = '3plus' |
| dose2_date | Donner la date approximative de la deuxième dose. | Si le jour est inconnu, sélectionner le 15 du mois. Si le mois est inconnu, sélectionner le mois de juin. | ___/___/______ | ${doses} = '2' or ${doses} = '3plus' |
| dose2_dist | Pour la deuxième dose, préciser le type de distribution |  | M, Campagne de masse  C, CATI  X, Autre  U, Inconnu | ${doses} = '2' or ${doses} = '3plus' |
| dose2_raison_nonvax | Pour la deuxième dose, raison de la non-vaccination |  | 1, Pas au courant de la campagne  2, Les vaccinateurs ne sont pas passés à la parcelle  3, Etait absent lorsque les vaccinateurs sont passés  4, Etait âgé moins d'un an  5, Etait enceinte  6, Refus pour cause de tradition  7, Refus pour cause de religion  8, La vaccination est dangereuse  9, Mauvaises expériences avec d'autres vaccins dans le passé  10, Il n'y avait pas assez de vaccin  11, N'a pas eu le temps  12, Etait malade (à la maison)  13, Etait hospitalisé  14, Le goût est mauvais  15, N'habitait pas dans une zone ciblée  16, Aucune explication | ${doses} = '0' or ${doses} = '1' |
| zds2 | La deuxième dose a été reçue dans quelle Zone de Santé ? |  | Goma, Goma  Karisimbi, Karisimbi  Kirotshe, Kirotshe  Nyiragongo, Nyiragongo  hors goma, Autre (hors Goma) | ${doses} = '3plus' or ${doses} = '2' |
| ads2 | Pour la deuxième dose, préciser là où la dose a été reçue | A Goma, choisir l'aire de santé. Hors Goma, choisir la province. | Liste des aires de santé de la ville de Goma | ${doses} = '2' or ${doses} = '3plus' |
| agglom2 | Si la deuxième dose a été reçu hors Goma, donner le nom de la Zone de santé ou l'agglomération la plus proche de la site où la vaccination a été reçu. |  | ________________ | ${zds2} = 'hors goma' |
| dose3_vax | Pour la troisième ou dernière dose d'OCV reçue depuis janvier 2019, cette personne est: |  | V, Vacciné, confirmation verbale  C, Vacciné, confirmé avec la carte  U, Inconnu | ${doses} = '3plus' |
| dose3_date | Donner la date approximative de la troisième ou dernière dose. | Si le jour est inconnu, sélectionner le 15 du mois. Si le mois est inconnu, sélectionner le mois de juin. | ___/___/______ | ${doses} = '3plus' |
| dose3_dist | Pour la troisième ou dernière dose, préciser le type de distribution |  | M, Campagne de masse  C, CATI  X, Autre  U, Inconnu | ${doses} = '3plus' |
| dose3_raison_nonvax | Pour la troisième ou dernière dose, raison de la non-vaccination |  | 1, Pas au courant de la campagne  2, Les vaccinateurs ne sont pas passés à la parcelle  3, Etait absent lorsque les vaccinateurs sont passés  4, Etait âgé moins d'un an  5, Etait enceinte  6, Refus pour cause de tradition  7, Refus pour cause de religion  8, La vaccination est dangereuse  9, Mauvaises expériences avec d'autres vaccins dans le passé  10, Il n'y avait pas assez de vaccin  11, N'a pas eu le temps  12, Etait malade (à la maison)  13, Etait hospitalisé  14, Le goût est mauvais  15, N'habitait pas dans une zone ciblée  16, Aucune explication | ${doses} != '3plus' |
| zds3 | La troisième ou dernière dose a été reçue dans quelle Zone de Santé? |  | Goma, Goma  Karisimbi, Karisimbi  Kirotshe, Kirotshe  Nyiragongo, Nyiragongo  hors goma, Autre (hors Goma) | ${doses} = '3plus' |
| ads3 | Pour la troisième ou dernière dose, préciser là où la dose a été reçue | A Goma, choisir l'aire de santé. Hors Goma, choisir la province. | Liste des aires de santé de la ville de Goma | ${doses} = '3plus' |
| agglom3 | Si la troisième ou dernière dose a été reçu hors Goma, donner le nom de la Zone de santé ou l'agglomération la plus proche de la site où la vaccination a été reçu. |  | ________________ | ${zds3} = 'hors goma' |
| diarh | Avez-vous eu les diarrhées aqueuses graves depuis janvier 2019? | Au moins 3 selles liquides dans 24 heures | Oui, Oui  Non, Non  U, Inconnu | ${cons} = 'oui' |
| diag | Avez-vous déjà été positif ou diagnostiqué du choléra depuis janvier 2019? |  | Oui, Oui  Non, Non  U, Inconnu | ${diarh} = 'Oui' |
| diag_date | Si oui, date approximative du diagnostic |  | ___/___/______ | ${diag} = 'Oui' |
| vivait_jan2019 | La personne vivait-elle dans cette même aire de santé depuis janvier 2019? |  | Oui, Oui  Non, Non  U, Inconnu |  |
| date_demenagt | Si non, date approximative de déménagement dans cette aire de santé |  | ___/___/______ | ${vivait_jan2019} = 'Non' |
| zds4 | Préciser la zone de santé où cette personne vivait le plus récemment avant cette aire de santé? |  | Goma, Goma  Karisimbi, Karisimbi  Kirotshe, Kirotshe  Nyiragongo, Nyiragongo  hors goma, Autre (hors Goma) | ${vivait_jan2019} = 'Non' |
| ads4 | Préciser où cette personne vivait le plus récemment avant cette aire de santé? |  | Liste des aires de santé de Goma | ${vivait_jan2019} = 'Non' |
| agglom_avant | Si la personne habitait hors Goma, donner le nom de la Zone de santé ou l'agglomération la plus proche de là où elle habitait avant. |  | _________________ | ${zds4} = 'hors goma' |

**Supplementary Table 3: Stated primary reason for non-vaccination with OCV for the first dose among unvaccinated participants, by sex, for participants in the cholera vaccine coverage survey in the Health Zones of Goma, Karisimbi and Nyiragongo, North Kivu, DRC, August 2022.**

| **Reason** | **Females (% of total participants)** | **Males (% of total participants)** | **Total^1^** |
| --- | --- | --- | --- |
| Not aware of the campaign | 225 (39.4) | 251 (34.8) | 508 (36.8) |
| Absent when the vaccinators came | 136 (21.0) | 198 (27.5) | 337 (24.4) |
| Vaccinators did not come | 73 (11.3) | 90 (12.5) | 164 (11.9) |
| Under one year of age | 63 (9.7) | 71 (9.8) | 135 (9.8) |
| Believe vaccine is dangerous | 33 (5.1) | 30 (4.2) | 63 (4.6) |
| Refused due to beliefs related to tradition | 32 (4.9) | 32 (4.4) | 64 (4.6) |
| No reason given | 24 (3.7) | 33 (4.6) | 57 (4.1) |
| Did not live in a targeted zone | 23 (3.6) | 16 (2.2) | 39 (2.8) |
| Negative experience in the past | 12 (1.9) | 21 (2.9) | 34 (2.5) |
| Refused due to beliefs related to religion | 6 (0.9) | 6 (0.8) | 13 (1.0) |
| Refused due to pregnancy | 8 (1.2) | 0 (0.0) | 9 (0.6) |
| The vaccine tastes bad | 2 (0.3) | 1 (0.1) | 3 (0.2) |
| Sick at home at time of campaign | 2 (0.3) | 0 (0.0) | 2 (0.0) |
| Hospitalised at time of campaign | 1 (0.2) | 0 (0.0) | 1 (0.0) |
| Not enough vaccine | 0 (0.0) | 0 (0.0) | 0 (0.0) |
| Not enough time | 0 (0.0) | 0 (0.0) | 0 (0.0) |

^1^Includes those of unknown sex

1. Henderson RH, Sundaresan T. Cluster sampling to assess immunisation coverage: A review of experience with simplified sampling methodology. Bulletin of the World Health Organization 1982(60):253-60 [↑](#footnote-ref-2)
2. <http://www.openepi.com/Menu/OE_Menu.htm> (accessed June 16, 2015) [↑](#footnote-ref-3)
